# Successful Repair of Esophageal Atresia with Tracheoesophageal Fistula and Interrupted Inferior Vena Cava: A Rare Case Report

**DOI:** 10.1055/a-2448-3530

**Published:** 2024-11-18

**Authors:** Xiao Long Mu, Junqiu Wang

**Affiliations:** 1 Department of Pediatrics, Pediatric Surgery, Taihe Hospital, Shiyan, Hubei, People's Republic of China; 2 Department of Dermatology, Taihe Hospital, Shiyan, Hubei, People's Republic of China

**Keywords:** esophageal atresia, tracheoesophageal fistula, interrupted inferior vena cava

## Abstract

Esophageal atresia (EA) with tracheoesophageal fistula (TEF) is a congenital anomaly that can present complex surgical challenges, especially when accompanied by rare vascular conditions like an interrupted inferior vena cava (IVC). The division of the azygos vein is a common part of TEF repair, but in the presence of an interrupted IVC, this can lead to life-threatening complications. We report the case of a newborn diagnosed with EA, TEF, and interrupted IVC, successfully treated through thoracotomy. This case underscores the importance of prenatal and postnatal imaging to diagnose vascular anomalies prior to TEF repair, ensuring the preservation of the azygos vein to prevent fatal outcomes. Raising awareness of this rare association is crucial to optimizing surgical planning and outcomes.

## Introduction

Esophageal atresia (EA) is a congenital abnormality of the esophagus that is caused by incomplete embryonic compartmentalization of the foregut. EA commonly occurs with a tracheoesophageal fistula (TEF). 1 The overall worldwide prevalence of EA as calculated from national and international databases for congenital anomalies is 2 to 4 per 100,000 births. 1 Interrupted inferior vena cava (IVC) is a rare disease. According to postnatal series, interrupted IVC with azygos or hemiazygos continuation is a rare disorder occurring in 0.6 to 3% of the population with congenital heart disease. 2 Therefore, the association of interrupted IVC, EA with TEF, is exceedingly rare. EA and TEF accompanied by interrupted IVC that underwent successful repair in a few pediatric cases has been described. 1 3 Before undertaking TEF surgery, most authors advise dividing the azygos vein. 1 4 References do not routinely recommend checking for an interrupted IVC preoperatively. 1 5 However, dividing the azygos vein in the presence of an interrupted IVC may result in death. The aim of this study was to analyze the clinical and radiological features of EA and TEF accompanied by interrupted IVC in newborns. It emphasizes the importance of diagnosing interrupted IVC before undertaking TEF surgery and raises awareness of the importance of diagnosing vascular anomalies prior to EA and TEF repair ( Supplementary Material 1 , available in the online version). 4 5

## Case Report

*Patient history:* A 2.85-kg female infant was delivered at 39 weeks of gestation by natural labor (November 19, 2020), with a 1-minute Apgar score 10 points. Maternal health in good. A fetal echocardiogram performed at 35 weeks demonstrated EA and an interrupted IVC with azygos continuation to the right-sided superior vena cava. A postnatal echocardiogram confirmed the interrupted IVC, which does not involve the hepatic segment of the IVC, and light cardiac anomalies with small atrial septal defect and mildly tricuspid regurgitation.

**Fig. 1:**
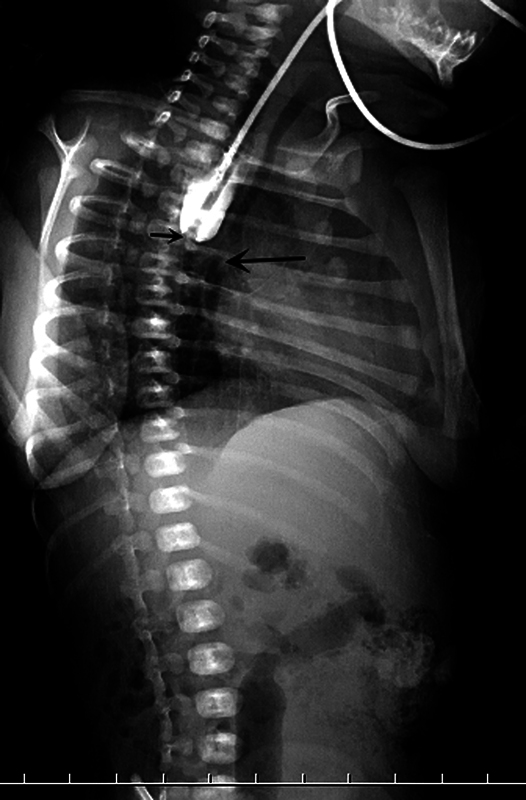
Chest radiograph on lateral view showed the orogastric tube inserted at the proximal esophageal pouch ( *short arrow* ) with distal bowel gas, esophageal atresia with distal tracheoesophageal fistula ( *long arrow* ).

*Diagnosis:* The patient was intubated at birth but resistance was noted during orogastric tube insertion. Chest X-ray revealed ( Fig. 1 ) the gastric tube at the upper pouch of the esophagus with the presence of intestinal gas, which is a classic feature of type C EA. Thoracotomy repair of EA was scheduled for the next day following stabilization.

*Surgical procedure:* The patient was positioned in a semi-prone position with the right chest slightly elevated. She was put on conventional ventilation without single lung ventilation. The patient underwent right intrapleural thoracotomy, based on the echocardiographic findings of an interrupted IVC and azygos continuation to the superior vena cava. The pediatric surgeon preserved the azygos vein connection at the time of the TEF repair to prevent obstruction of the venous return from the lower body. Through a right intrapleural thoracotomy, 5–0 Monocryl was used as anastomotic suture material and end-to-end esophageal anastomosis. All operations were performed by three qualified pediatric surgeons. A 10-Fr chest drain was inserted at the end of the procedure. The operation was uneventful and completed in 145 minutes.

**Fig. 2:**
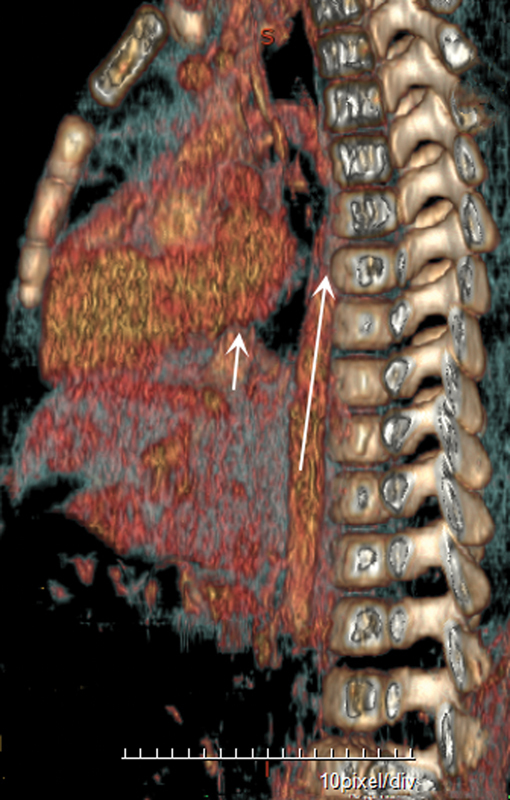
Contrast-enhanced computed tomography on sagittal view showed a hepatic segment of the inferior vena cava draining hepatic veins ( *short arrow* ) and not continuing further caudally. Iliac and renal veins drained via the distended azygos vein within the abdomen, entering the thorax and draining into the superior vena cava within the thorax ( *long arrow* ).

*Postoperative care:* After surgery, the patient was managed in the neonatal intensive care unit according to our usual protocol. The patient was extubated successfully on day 4 after surgery. She was discharged home at the age of 15 days. At 6 months of age, enhanced computed tomography (CT) showed ( Fig. 2 ) the hepatic segment of the IVC draining hepatic veins and not continuing further caudally. Iliac and renal veins drained via the distended azygos vein within the abdomen, entering the thorax and draining into the superior vena cava within the thorax, and by 12 months of age her small atrial septal defect, measured by Doppler echocardiography, was normal. At the follow-up visit until April 5, 2024, she was doing well, she had good weight gain with full oral feeding, and no clinical gastroesophageal reflux.

## Discussion

Interrupted IVC with azygos or hemiazygos vein continuation is a rare congenital anomaly. 2 It usually involves the hepatic segment of the IVC. According to a postnatal series, its estimated prevalence is 0.15%. 2 EA with or without TEF is the most common anomaly of the esophagus (incidence of 2–4/10,000 births). 1 The infrahepatic interruption of the IVC with azygos and hemiazygos continuation is a rare finding especially when it is associated with EA and TEF. 2 6 Interrupted IVC is associated with congenital heart disease in approximately 85% of cases, and frequently with the polysplenia syndrome. Our patient demonstrated light cardiac anomalies with small atrial septal defect, without polyspenia. 2 3 7 To the best of our knowledge, there has only been one report of this combination in a patient who survived surgery and continues to do well, 8 but there are few studies analyzing the radiological features of EA and TEF accompanied by interrupted IVC. This study analyzed the clinical and radiological features of EA and TEF accompanied by interrupted IVC, reported the successful outcome of the rare presentation of EA with TEF and an interrupted IVC, and emphasized that all EA patients should undergo a detailed evaluation of IVC during preoperative assessment.

The most commonly involved segment of interrupted IVC is the hepatic segment. It is due to the failure of the development of the hepatocardiac canal during the embryo, in its 10 to 15 mm length. 2 This patient's infrahepatic part was atretic; the iliac and renal veins drained via the distended azygos vein within the abdomen, entering the thorax and draining into the superior vena cava. IVC interruption can be diagnosed through prenatal or postnatal imaging. Noninvasive imaging modalities such as contrast-enhanced CT and magnetic resonance imaging are the most reliable methods for identification of these anomalies in an asymptomatic patient, and contrast-enhanced CT scan and venography can be helpful. Note was made of interrupted IVC, which drained only the hepatic veins before entering the right atrium. 9 The infrahepatic IVC continues as the azygos vein. Recognizing this venous anomaly is important for the pediatric surgeon, especially for conditions such as a paracardiac or mediastinal mass on chest radiography.

Although EA can be diagnosed antenatally, most patients (>90%) are diagnosed after birth. EA is prenatally diagnosed in a minority of cases and is usually only suspected on the basis of the presence of indirect or direct signs on ultrasonography. MRI with dynamic sequence and biochemical evaluation of the amniotic fluid have been developed to help in the diagnosis of EA. 5 6 10

Different surgical techniques are available. The optimal approach is dependent on the type of EA. Division of the azygos has been part of the TEF repair since Cameron Haight's report of the first successful procedure. 6 However, references do not routinely recommend checking for an interrupted IVC preoperatively. Dividing the azygos vein in the presence of an interrupted IVC may result in death. 9 10 Some suggest inspecting the size of the azygos vein prior to division, as a large azygos vein may be consistent with an interrupted IVC. 9 10 11 Patients born with EA should ideally be evaluated in a multidisciplinary team consisting of a pediatric surgeon, a gastroenterologist, a pulmonologist, and an otolaryngologist.

For TEF repair, it is important to be aware of the patients' vascular anatomy before surgery. 3 6 12 Accurate diagnosis of an abnormal IVC position and course can help the pediatric surgery expert in surgical planning, avoid unnecessary treatment measures, and prevent accidental injury caused by complications.

In conclusion, this case highlights the complexity of managing EA with TEF in the presence of an interrupted IVC. Early and accurate diagnosis of vascular anomalies through prenatal or postnatal imaging is essential for optimal surgical planning and avoiding potentially fatal complications. The preservation of the azygos vein during repair was critical in ensuring a successful outcome. This report emphasizes the importance of multidisciplinary care and comprehensive imaging in rare congenital cases to improve patient survival and outcomes.

## Notes

Importance for a Pediatric Surgeon For EA and TEF repair, it is important to be aware of the patients' vascular anatomy before surgery. Echocardiographic determination of both the aortic arch laterality and the status of the IVC and azygos vein proved helpful for the pediatric surgeon's approach to TEF repair.
