# Supplementary material for: Successful Repair of Esophageal Atresia with Tracheoesophageal Fistula and Interrupted Inferior Vena Cava: A Rare Case Report
Source: European J Pediatr Surg Rep. 2024 Nov 18;12(1):e77–80. doi: 10.1055/a-2448-3530 (PMC11869387; doi:10.1055/a-2448-3530)
Supplement: Supplementary file 1 — Supplementary Material [file 10-1055-a-2448-3530-s2024040752cr.ppt]

## Slide 1
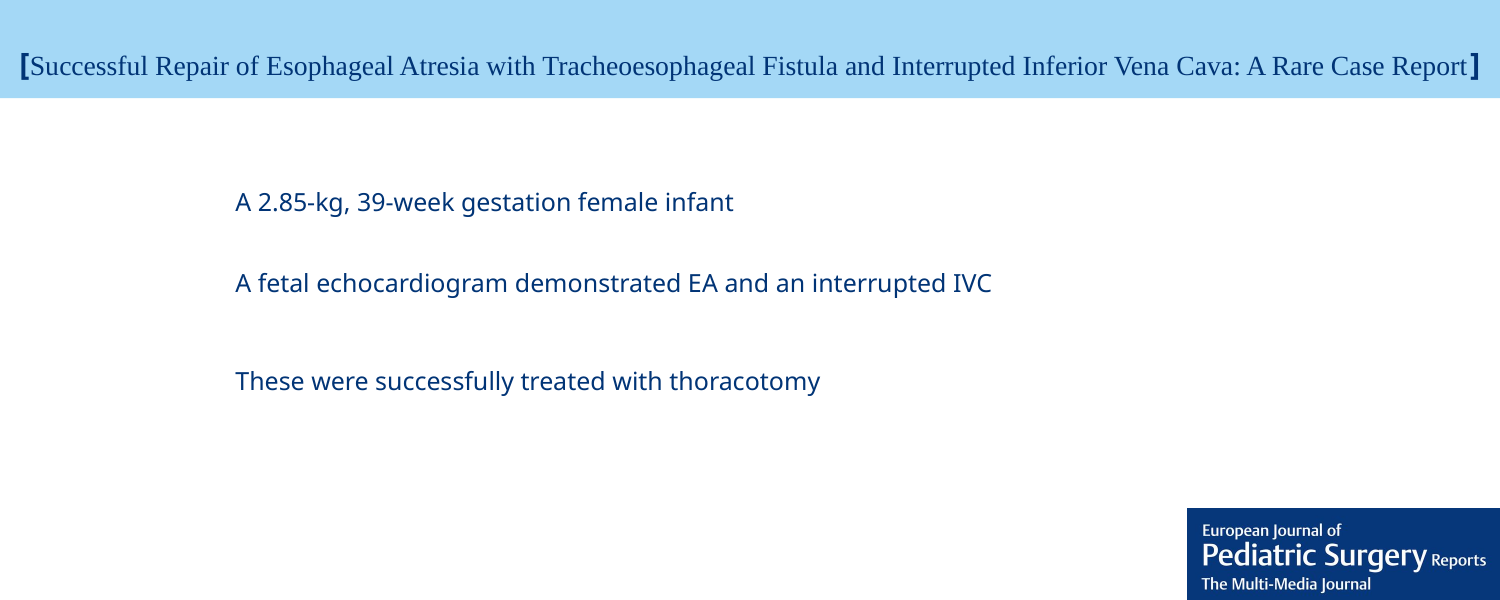

[Successful Repair of Esophageal Atresia with Tracheoesophageal Fistula and Interrupted Inferior Vena Cava: A Rare Case Report]
A 2.85-kg, 39-week gestation female infant
A fetal echocardiogram demonstrated EA and an interrupted IVC
These were successfully treated with thoracotomy

## Slide 2
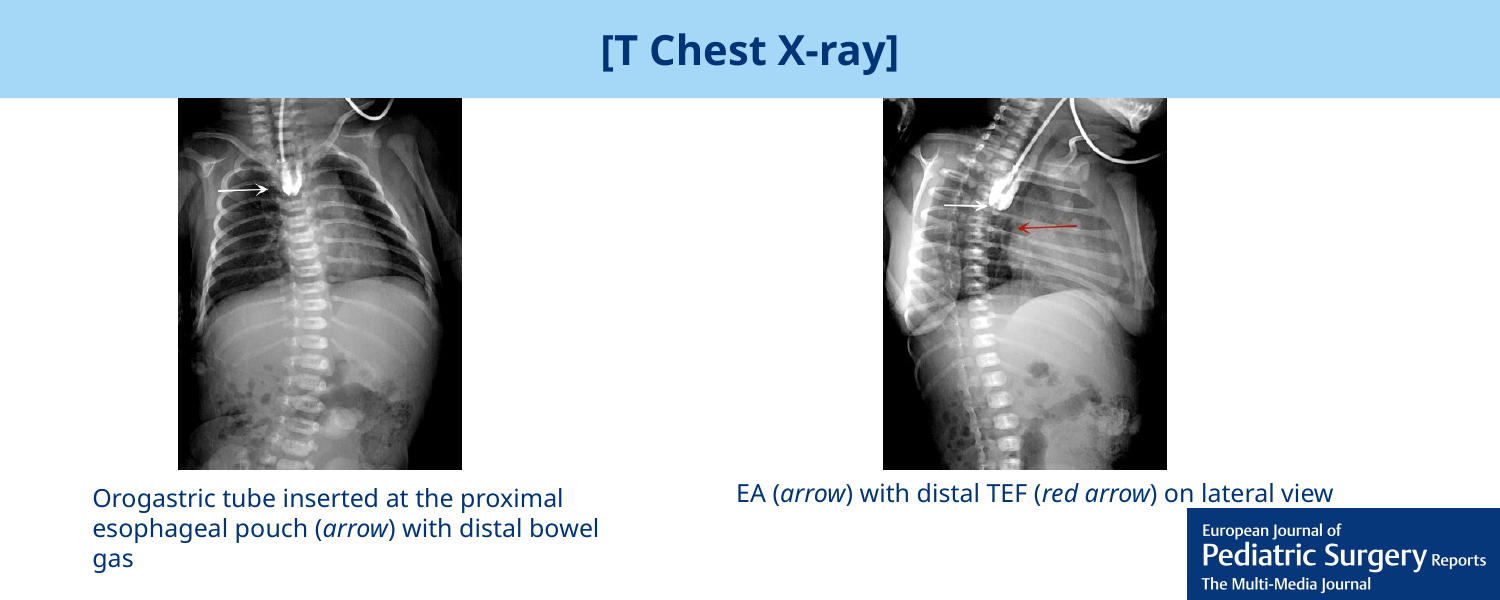

[T Chest X-ray]
EA (arrow) with distal TEF (red arrow) on lateral view
Orogastric tube inserted at the proximal esophageal pouch (arrow) with distal bowel gas

## Slide 3
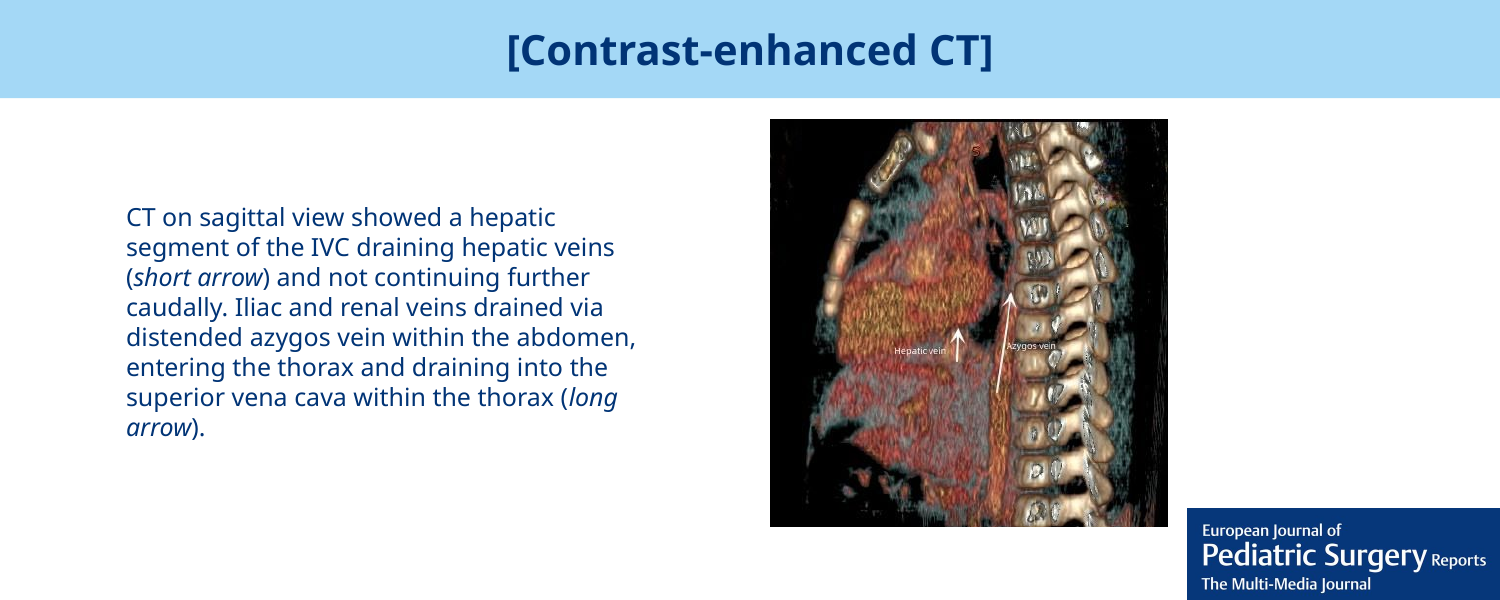

[Contrast-enhanced CT]
CT on sagittal view showed a hepatic segment of the IVC draining hepatic veins (short arrow) and not continuing further caudally. Iliac and renal veins drained via distended azygos vein within the abdomen, entering the thorax and draining into the superior vena cava within the thorax (long arrow).

## Slide 4
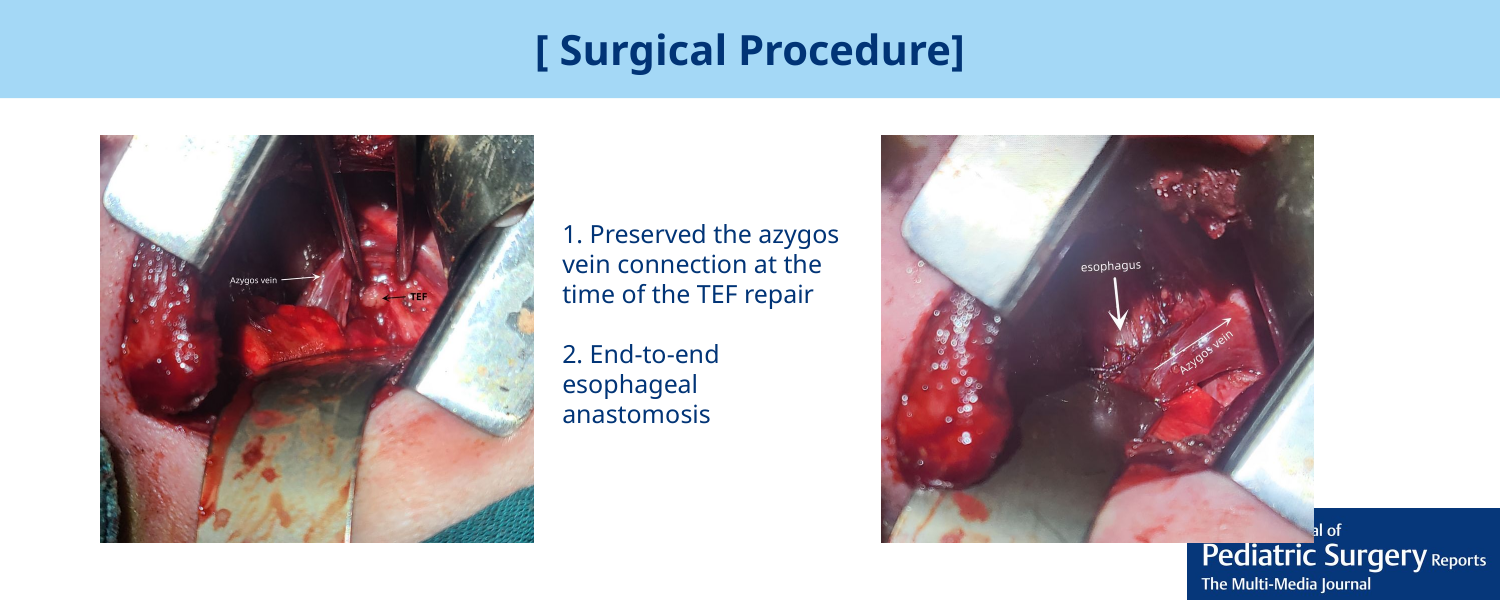

[ Surgical Procedure]
1. Preserved the azygos vein connection at the time of the TEF repair
2. End-to-end esophageal anastomosis
